# An improved dimensionality reduction method for meta-transcriptome indexing based diseases classification

**DOI:** 10.1186/1752-0509-6-S3-S12

**Published:** 2012-12-17

**Authors:** Yin Wang, Yuhua Zhou, Yixue Li, Zongxin Ling, Yan Zhu, Xiaokui Guo, Hong Sun

**Affiliations:** 1 College of Life Science and Biotechnology, Shanghai Jiaotong University, 800 Dongchuan Road, Shanghai 200240, China; 2 Key Laboratory of Systems Biology, Shanghai Institutes for Biological Sciences, Chinese Academy of Sciences, Shanghai 200031, China; 3 Shanghai Center for Bioinformation Technology, Shanghai 200235, China; 4 Department of Medical Microbiology and Parasitology, Institutes of Medical Sciences, Shanghai Jiao Tong University School of Medicine, Shanghai 200240, China; 5 State Key Laboratory for Diagnosis and Treatment of Infectious Diseases, the First Affiliated Hospital, College of Medicine, Zhejiang University, Hangzhou, Zhejiang 310003, China; 6 Department of Cardiology, Gansu Provincial Hospital, Lanzhou 730000, China

## Abstract

**Background:**

Bacterial 16S Ribosomal RNAs profiling have been widely used in the classification of microbiota associated diseases. Dimensionality reduction is among the keys in mining high-dimensional 16S rRNAs' expression data. High levels of sparsity and redundancy are common in 16S rRNA gene microbial surveys. Traditional feature selection methods are generally restricted to measuring correlated abundances, and are limited in discrimination when so few microbes are actually shared across communities.

**Results:**

Here we present a Feature Merging and Selection algorithm (FMS) to deal with 16S rRNAs' expression data. By integrating Linear Discriminant Analysis method, FMS can reduce the feature dimension with higher accuracy and preserve the relationship between different features as well. Two 16S rRNAs' expression datasets of pneumonia and dental decay patients were used to test the validity of the algorithm. Combined with SVM, FMS discriminated different classes of both pneumonia and dental caries better than other popular feature selection methods.

**Conclusions:**

FMS projects data into lower dimension with preservation of enough features, and thus improve the intelligibility of the result. The results showed that FMS is a more valid and reliable methods in feature reduction.

## Background

The biogeography of microbiota in the human body are linked intimately with aspects of host metabolism, physiology and susceptibility to disease [1,2]. Previous studies have identified that dysbiosis of the distribution or infection of pathogenic microbiota would lead to some human diseases, such as pneumonia [3], dental caries [4], cutaneous disease [5], or other disease [6,7]. Characterization of the abundant and rare microbiota represents essential groundwork to human's health [3,8]. Knowledge of the human microbiome has been expanded greatly by various techniques such as 16S rRNA gene sequencing and metagenomics, etc. Gene expression sequencing enables the simultaneous measurement of the expression levels of thousands of genes. Like gene selection, the curse of dimensionality also applies to the problem of microbiota classification [9,10].

The ability to successfully distinguish between disease classes using gene expression data is an important aspect of approaches to disease classification, the discrimination methods include nearest-neighbor, linear discriminant analysis, and classification trees etc [11]. The nature of gene expression data and its acquisition means that it is subject to the curse of dimensionality, the situation where there are vastly more measurable features (genes) than there are samples. Dimension reduction methods are much used for classification or for obtaining low-dimensional representations of datasets. Traditionally, there are two types of methods used to reduce dimensionality. One is feature selection and the other is feature transformation [12]. Feature selection techniques do not alter the original representation of the features, but merely select a subset of features derived from the large set of profiles. Three kinds of feature selection were widely used: filter methods, wrapper methods and embedded methods [13]. However, most of existing feature selection methods reduce a feature space of high dimensionality into a manageable one at the cost of losing the relationship between different features.

Contrasted with feature selection, feature transformation methods create a new feature space with an optimal subset of predictive features measured in the original data. Some traditional feature transformation methods, such as principal component analysis (PCA) and linear discriminant analysis (LDA), output a combination of original features. PCA converts a set of possibly correlated variables into a set of orthogonal factors that efficiently explain the variance of the observations. LDA transforms original features to k-1 dimensions if there are k categories of training data. These traditional methods are fast and easy to compute, but there are some weakness [14], like that not all the discrimination vectors obtained are useful in pattern classification and that features of different dimensions are overlapping, thus it is often difficult to interpret the results.

Previous surveys showed that taxon relative abundance vectors from 16S rRNA genes expression provide a baseline to study the role of bacterial communities in disease states [15-17]. However, high levels of sparsity are common in 16S rRNA gene microbial surveys, presenting the fundamental challenge for their successful analysis. Identifying which microbes will produce good discrimination remains challenging when so few microbes are actually shared across communities. Besides, in a typical study of microbiota, 16S rRNAs' expression level of different samples might be redundant. Traditional feature selection methods are generally restricted to measuring correlated abundances, and are limited in their ability to maintain the information due to the removal of redundant features. In microbiota analysis, it is critical to preserve enough features to improve the intelligibility with minimized classification error rate and effectively reduced feature dimension simultaneously. To solve these problem, we introduced an improved Feature Merging and Selection algorithm (FMS in short) to identify combinations of 16S rRNA genes that give the best discrimination of sample groups. FMS extracts essential features from the high dimension feature space, then, an efficient classifier is employed with a lower classification error rate, to project data into lower dimension and preserve enough features and thus improve the intelligibility of the result. The performances were tested by 16S rRNAs' expression datasets of pneumonia patients and that of dentes cariosus patients.

## Results

### Feature Merging and Selection algorithm

Two statistics methods were considered to handle the continuous and sparse data of 16S rRNAs' expression levels. Fisher statistic was used to test the classification ability of features and Pearson Correlation Coefficient was used to describe the redundancy between features. We developed a new method called Feature Merging and Selection algorithm, which combined Linear Discriminant Analysis (LDA) method to learn linear relationship between different features. Classical LDA requires the total scatter matrix to be nonsingular. However, in gene expression data analysis, all scatter matrices in question can be singular since the data points are from a very high-dimensional space and in general the sample size does not exceed this dimension. To deal with the singularity problems, classical LDA method was modified in a way that an unit diagonal matrix with small weights was added to the within-class scatter matrix. The procedure continued until the remaining matrix eventually became nonsingular.

**Figure 1:**
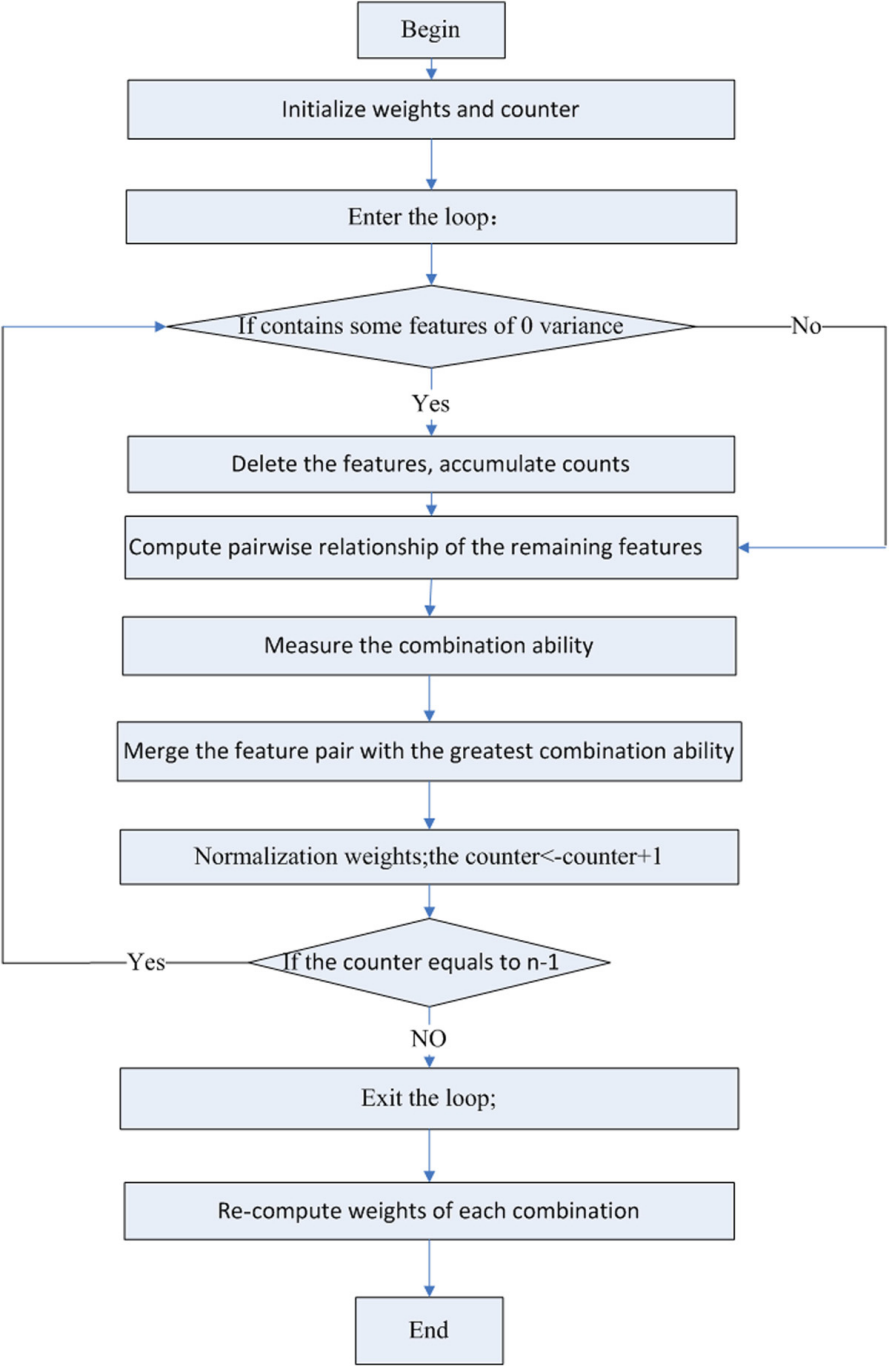
**FMS algorithm flowchart**.

FMS algorithm consists of two parts: feature merging and feature deletion. Feature merging is the main part of the algorithm. The procedure is described below (see Figure 1):

Step 1: Initialization: set weights of all the features to 1 and the counter to 0; label each feature from 1 to n, n is the total number of features.

Step 2: Loop from step 2 to step 7 until the counter equals to n-1.

Step 3: Delete features of zero variance, and add the total number of deleted features to the counter.

Step 4: Compute pairwise relationship of the remaining features using modified LDA, and preserve the combination features with maximal Fisher statistics. The Fisher statistics is defined as $\frac{\underset{k}{\sum }n_{k}{\left(m_{k}-m\right)}^{2}/\left(K-1\right)}{\underset{k}{\sum }\left(n_{k}-1\right){\sigma_{k}}^{2}/\left(n-K\right)}$, where K is the total number of classes, n is the size of all the samples, *n_k _*is the size of the *k*th class, *m_k _*is the mean value of the sample within the *k*th class, m is the mean value of all the samples, and $\sigma_{k}^{2}$ is the variance of the *k*th class.

Step 5: Measure the combination ability by combining Fisher statistics method and Pearson correlation coefficient methods, and calculate the merging value = (new value of Fisher statistics)*(Pearson Correlation Coefficient)/(geometric mean values of Fisher statistics of the original features).

Step 6: Select and merge the feature pair with the greatest merging value, save the original labels, and multiply the weight by previously trained weight.

Step 7: Normalize the weight; add 1 to the counter.

Step 8: Re-compute the weight of each combination using LDA until the original feature number is less than two. Preserve the combination with maximal Fisher statistics value and normalize the weights.

After feature merging, the resulting combinations reveals the relationship between the original features. With more features deleted, linear bias is getting greater, but variance is getting lower; and vice versa. To compromise between the bias and variance criteria, we selected the dimension reduction ratios by 5-fold proportional cross validation [12,18]. The whole data was partitioned into two parts as training data and test data. Training data was used for feature merging to learn relationship between features and output combination of features. Test data was used to estimate the error rate of feature merging. If there exists equal error rates among two or more feature merging performances, the one with the largest merging degree will be left to obtain lower dimension feature vectors.

To simplify the model, features were deleted based on the resulting combinations after feature merging and cross validation. Values of fisher statistics were multiplied by the weight of each combination. Features were sorted in ascending order by absolute value of their weights and were deleted one by one, and the error rate were got by 5-fold proportional cross validation. For those classification performances with equal error rates, the decision was then made to preserve the resulting combination with lower dimensions or less number of features. Unimportant features were thus deleted to simplify the model. In summary, FMS determine the final dimensionality and thus the optimal number of features which yields the lowest error rate got by cross validation. FMS algorithm is a dimensionality reduction method and should be used with combination of a classifier.

Fisher method has a high classification ability on datasets with low noise, but its performance can be reduced because of the noisy data. To address the weakness of fisher method when dealing with noisy data, mutual information method was used for feature deletion instead of Fisher statistic method. Under Occam's razor [19], we considered classification combinations with lowest dimension as the simplest result. We calculated the error rate plus penalty with each dimension as a criteria for feature selection [13], and selected the first m performances with the lowest value, where m is log(N) and N is the original dimension. Weight of penalty was set as the range of the first m error rates divided by the range of relevance dimension. If the first t feature merging performances got same value of error rate plus penalty, then set m to log(N)+t-1. This method provided an alternative way to deal with noisy data.

### Examples of FMS algorithm

We first tested the FMS algorithm on the 16S rRNAs' expression profiles got from pneumonia samples belonged to three classes, 101 patients with hospital-acquired pneumonia (HAP), 43 patients with community-acquired pneumonia (CAP), and 42 normal persons as control [3]. We assigned the 16S rRNAs' expression profiles into the microbe taxonomy as 16S rRNA sequences are often conserved within a species and generally different between species. The expression data matrix was further expressed as percentage values of microbiota. Features with zero variance were deleted. The whole data was partitioned into two parts for training and testing the model. The training data included profiles from 71 cases of HAP, 32 cases of CAP and 30 cases of normal samples and the test data included profiles from 30 cases of HAP , 13 cases of CAP and 12 cases of normal samples. The training data was used for cross validation, and the test data was use to control the error rate. Five-fold proportional cross validation was performed on the training data to determine the degree of feature merging and feature deletion.

The feature merging algorithm was then performed on the whole training data based on the obtained degrees of feature merging and feature deletion, the output reflected the relationship between combinations of features. Then the classifier was used to produce a classification on test data, and error rate was obtained. K-nearest neighbor algorithm (kNN) and Support vector machine (SVM) are widely used tools for classification. SVM was selected as classifier along with the algorithm because of its lower error rate for the pneumonia training data. Four widely used feature selection methods, mRMR method [20], Information Gain method [21], χ^2 ^statistic [22] and Kruskal-Wallis test method [23] were used as controls to test the validity of FMS method.

Two types of classification were considered: three-class problem and two-class problem. The former outputs three classes, i.e. HAP, CAP and normal, the later outputs two classes, *i.e*. pneumonia (HAP and CAP) and normal. As SVMs are inherently two-class classifiers, therefore one-against-all decomposition technique was used to divide a three-class classification problem into two binary class ones. Normal samples were discriminated from pneumonia samples at first step, then HAP and CAP were discriminated. For the two class problem, the training data was imbalanced because of the lesser number of normal samples compared with pneumonia samples. Pneumonia samples were thus clustered into three subgroups [24], then each pneumonia subgroup was mixed with data from normal samples to form a training dataset. The model was trained on all mixed datasets. Each classification performance on test data gave a vote to each class.

**Figure 2:**
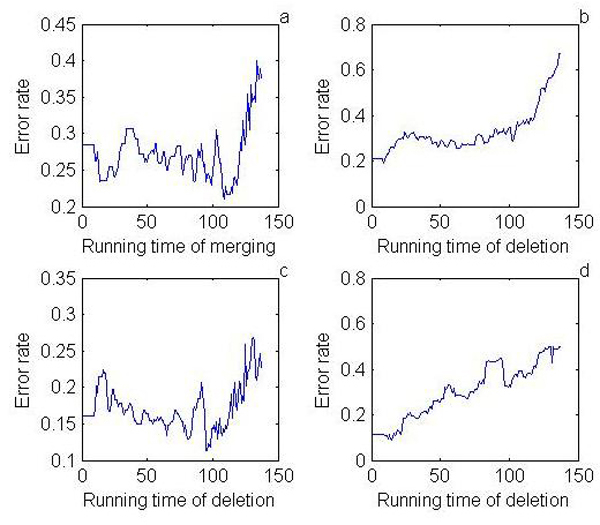
**Learning curves of FMS algorithm for feature merging in 3-class problem (a), feature deletion in 3-class problem (b), feature merging in 2-class problem (c) and feature deletion in 2-class problem (d)**.

For balanced training data, error rates obtained from the whole training data is suited to measure classification ability. However, it is not suitable for imbalanced data. Therefore, the mean error rate [25] of each class was used to measure the classification performance. The error rate of the *i*th category was calculated as $\frac{F_{i}}{T_{i}+F_{i}}$, where *T_i _*is the percentage of the *i*th category of samples with the correct label and *F_i _*is the percentage of the *i*th category of samples with wrong label [26]. The learning curves showed that the lowest error rate was achieved with 108 times feature merging performances and 8 deleted features in 3-class problem (Figure 2a, b), and 95 times feature merging performances and 13 deleted features in 2-class problem (Figure 2c, d).

Combined with either SVM or kNN classifier, FMS algorithm has the lowest mean error rate in both the 3-class and 2-class problems compared with four other widely used feature selection methods, *i.e*. mRMR method [20], Information Gain method [27] , **χ^2 ^**statistic [22] and Kruskal-Wallis test method [23]. Both in three-class and two-class problem, FMS algorithm reduced the dimension of the original data to a lower or close level compared with the other four commonly used feature deletion methods, and preserved enough features (Table 1 Table 2). ROC curve is the representation of the tradeoffs between sensitivity and specificity for various threshold values to define an abnormal test. ROC was constructed for each subset of features. The ROC curves showed that the optimal features determined by FMS, which were selected under the criteria of lowest error rate got by cross validation, reached high accuracy (~80%) with high sensitivity (~80%) (Additional file 1 Figure 1 and 2), and that high specificity were obtained as a whole, demonstrating the feature reduction quality of FMS. The results showed that combined with classifier the use of FMS algorithm output lower dimension combinations of features and achieved lower classification error rate. FMS combined with SVM classifier performed better in classification than combined with kNN classifier, therefore FMS combined with SVM was used to classify the 16S rRNAs' expression profile of pneumonia samples, and the classification results were used for further analysis.

**Table 1:** Classification ability on pneumonia data in 3-class problem

Method	Error rate		Dimension	Feature number	Note
				
	On training data	On test data			
svm/FMS	0.1895	0.2637	29	129	

svm/mRMR	0.2267	0.3103	38	38	

svm/KruskalWallis	0.1984	0.3816	107	107	

svm/InformationGain	0.2425	0.3684	28	28	

svm/χ2 statistic	0.2127	0.4308	125	125	

svm	0.2841	0.4017	137	137	

kNN/FMS	0.2013	0.3406	112	133	k = 1

kNN/mRMR	0.2635	0.3774	130	130	k = 1

kNN/KruskalWallis	0.2492	0.3795	134	134	k = 1

kNN/InformationGain	0.2635	0.3774	130	130	k = 1

kNN/χ2 statistic	0.2537	0.4128	124	124	k = 1

kNN	0.2635	0.3774	137	137	k = 1

**Table 2:** Classification ability on pneumonia data in 2-class problem.

Method	Error rate		Dimension	Feature number	Note
				
	On training data	On test data			
svm/FMS	0.0922	0.1279	42	123	

svm/mRMR	0.1313	0.1977	36	36	

svm/KruskalWallis	0.1081	0.1628	62	62	

svm/InformationGain	0.1456	0.186	54	54	

svm/χ2 statistic	0.1561	0.186	127	127	

svm	0.1611	0.1977	137	137	

kNN/FMS	0.1279	0.2393	20	130	k = 1

kNN/mRMR	0.2532	0.3372	54	54	k = 1

kNN/KruskalWallis	0.1861	0.3343	25	25	k = 4

kNN/InformationGain	0.2248	0.3256	107	107	k = 1

kNN/χ2 statistic	0.336	0.4535	107	107	k = 1

kNN	0.346	0.4419	137	137	k = 1

**Figure 3:**
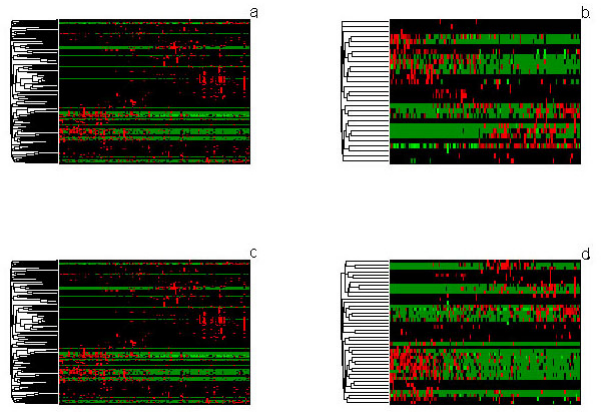
**The expression profiles of original pneumonia data for 3-class problem (a), data after treated by FMS for 3-class problem (b); original pneumonia data for 2-class problem (c) and data after treated by FMS for 2-class problem (d)**. Rows are microbiotas and columns are disease classes. From left to right are 30 normal, 32 CAP, 71 HAP samples for 3-class problem, and 30 normal 103 pneumonia samples for 2-class problem.

Heatmap is a frequently used matrix of pair-wise sample correlations in which anti-correlation or correlation is indicated by a color-scale, *e.g*. green to red. From the heatmap matrix of all original 16S rRNA's expression data (Figure 3a, c), similarities and differences between samples or genes are easily lost due to the large size of these visualizations. After feature extraction by FMS, the original space has been reduced to the space spanned by a few features, with data loss but retaining the most important variances (Figure 3b, d). The pair-wise display of samples indicates similarity in expression profiles much more clearly and with a high resolution after the dimensionality reduction.

**Figure 4:**
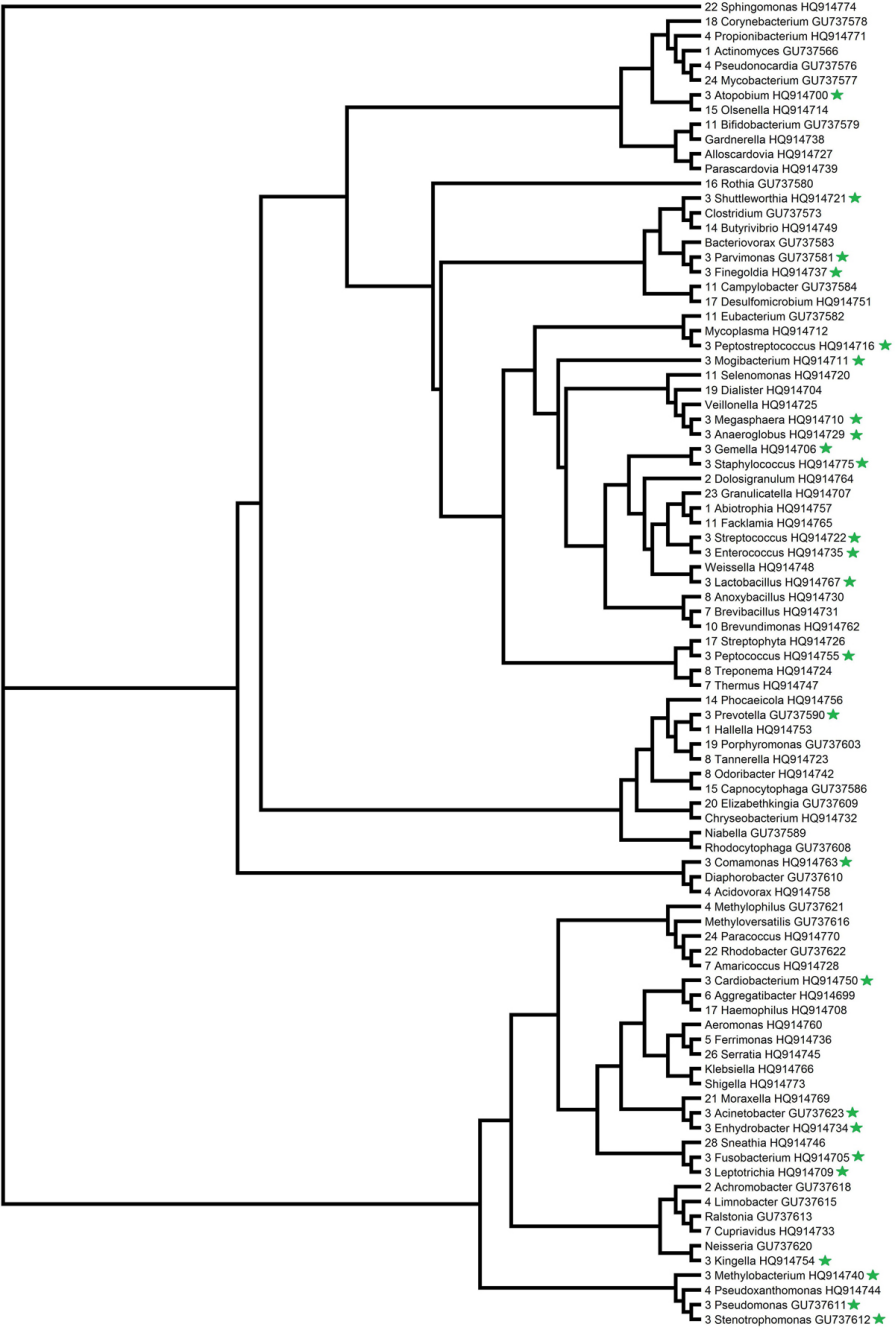
**Phylogenetic relationship of microbiota signatures in 3-class problem**. The microbiota signatures with best discrimination ability were labeled with green star.

**Figure 5:**
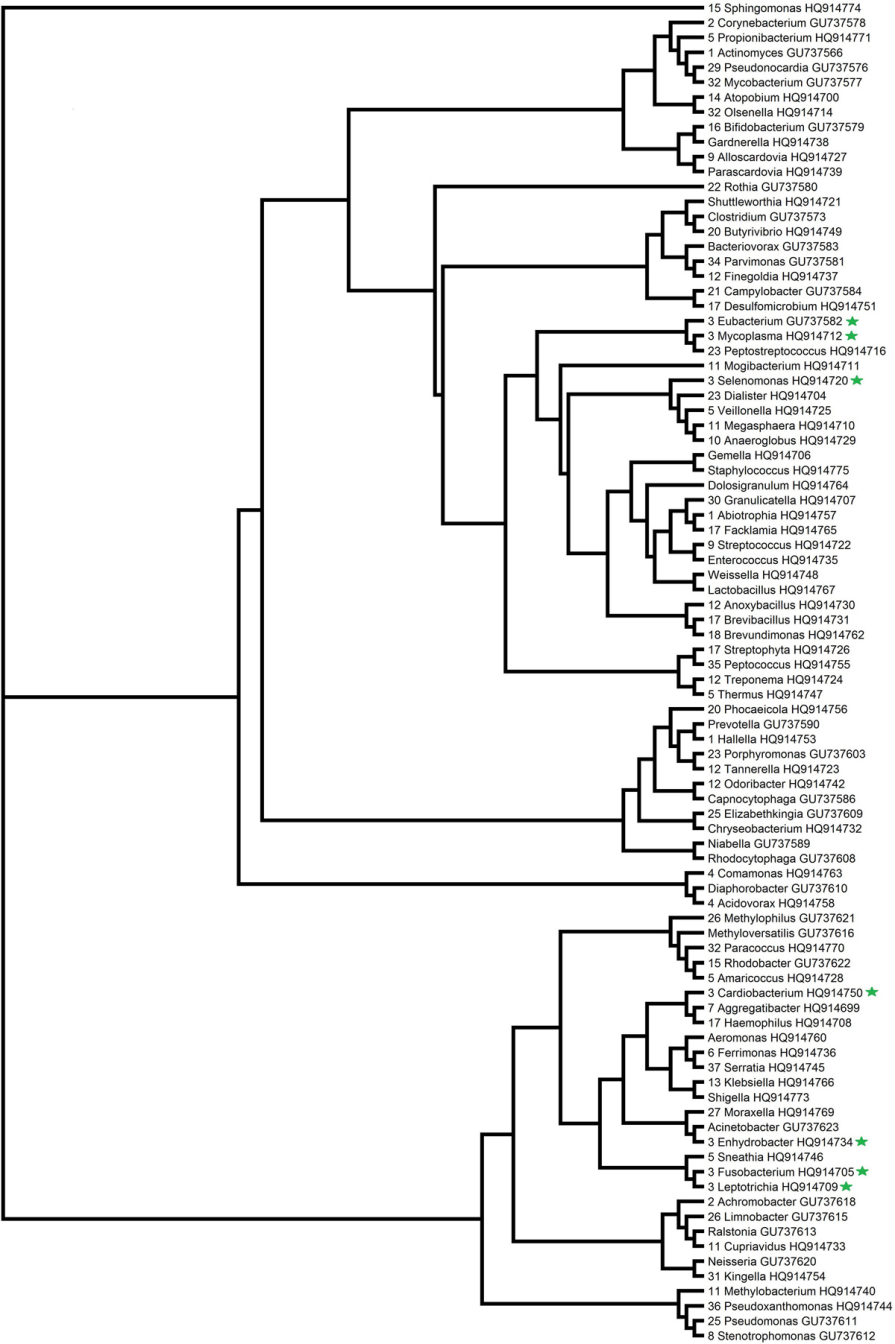
**Phylogenetic relationship of microbiota signatures in 2-class problem**. The microbiota signatures with best discrimination ability were labeled with green star.

Combinations of features were sorted by their Fisher statistics, which indicated the discrimination ability. The microbiota signatures with best discrimination ability enabled us to identify low- and high-risk patients with distinct pneumonia classes (Additional file 1 Table 1). The results showed that shuttleworthia characterized as a distinct indicator of pneumonia in three-class problem, and acidaminococcus in two-class problem. It has been previously observed that shuttleworthia and acidaminococcus are causes of pneumonia [28,29]. Of the top 20 genera suspiciously contributing to the hospital-associated pneumonia [3], about half were found in the resulting combination with best discrimination ability in three-class problem (Additional file 1 Table 1). FMS discriminates microbial signatures efficiently, which will enable improved disease classification. Phylogenetic trees were constructed based on the nucleotide sequences of microbiota 16S rRNAs. It is noteworthy that the microbiota signatures are dispersed in the phylogentic tree (Figure 4, 5), which indicates that the enormously diverse microbiota performs important functions for the host organism. FMS provides a combination of taxonomically wide set of microbiota signatures to evaluate agents' contribution to the infection.

FMS algorithm was also tested on 16S rRNAs' profiles form dental decay patients. These samples were collected from saliva and dental plaques separately. For the expression level of 16S rRNAs collected from dental plaques samples, the training data contains 23 dental decay patient samples and 20 normal samples and the test data contains 9 dental decay patient samples and 8 normal samples. For the expression level of 16S rRNAs collected from saliva samples, the training data contains 23 dental decay patient samples and 19 normal samples and the test data contains 10 dental decay patient samples and 8 normal samples. As these dental decay datasets are noisy, mutual information method was used for feature deletion instead of Fisher statistic method. When treating with the noisy data, the data showed that FMS also performed better than mRMR method [20] and Kruskal-Wallis test method [23] (Additional file 1 Table 2, 3).

## Conclusions

In this work, we introduced FMS algorithm to address the high level sparsity and redundancy problem of 16S rRNA genes microbial surveys, thereby identifying combinations of 16S rRNA genes that give the best discrimination of sample groups. FMS method has several distinct advantages and features that make it useful to researchers: 1) FMS reduces feature dimension with higher accuracy and preserves the relationship between different features as well, thus improve the intelligibility of the result. 2) FMS processes features into sets of combinations and performs more efficiently and meaningfully in distinguishing among classifications than the individual features, which is in line with the observation that particular combinations of specific bacteria are associated with individual symptoms and signs [30]. 3) FMS uses combined features on classification performance, which may compensate for the influence of individual features, thus provides more robust classification with higher accuracy and less variation. 4) Different from LDA, FMS classifies features into combinations, features of different combinations were not overlapping and the relationship between features were well preserved.

In conclusion, we developed a new feature merging and selection algorithm to deal with 16S rRNAs expression data in order to reduce feature dimensionality and retain enough important features. The improved method reserves some advantages of both LDA and other feature selection methods, and reduces dimensions much more effectively. As the classification examples showed, the FMS algorithm reduced dimensionality of the data effectively without losing important features, which made results more intelligible. FMS performed well and will be useful in human microbiome projects for identifying biomarkers for disease or other physiological conditions.

## Data and method

### Data

We got the 16S rRNAs' expression profiles of pneumonia patients from Zhou et al., [3], and 16S rRNAs' expression profiles of dental decay patients from Ling et al, .[4]. The set of 16S rRNAs' sequences, which were used for constructing the phylogenetic trees, were downloaded from NCBI website (ID: GU737566 to GU737625, and HQ914698 to HQ914775) (http://www.ncbi.nlm.nih.gov). After removing redundant sequences, a total of 90 microbe species were used for phylogenetic analysis.

### Linear Discriminant Analysis

Linear Discriminant Analysis(LDA) is a typical variable transformation method to reduce dimensions [31]. The key of LDA is to maximize the Rayleigh quotient: $\text{J}\left(\text{W}\right)=\frac{\alpha^{T}S_{B}\alpha }{\alpha^{T}S_{W}\alpha }$, where $S_{B}=\underset{k}{\sum }\left(n_{k}-1\right)\sum \left(m_{k}-\text{m}\right){\left(m_{k}-\text{m}\right)}^{\prime }/\left(K-1\right)$, is the "between classes scatter matrix", and $S_{W}=\overset{K}{\underset{k}{\sum }}\left(y-m_{k}\right){\left(y-m_{k}\right)}^{\prime }/\left(\text{N}-\text{K}\right)$, is the "within classes scatter matrix". K is the number of classes, and *n_k _*is the number of the samples within the kth class. *m_k _*is the mean value of the sample within the kth class, and m is the mean value of all the samples.

LDA method can find a direction which maximizes the projected class means and while minimizing the classes variance in this direction. To avoid *S_W _*become singular matrix, we added unit matrix with small weights to *S_W _*in each loop until *S_W _*became non-singular. The program can be downloaded from http://www.mathworks.com/matlabcentral/fileexchange/29673-lda-linear-discriminant-analysis/content/LDA.m

### Support vector machine algorithm

Support vector machine (SVM) algorithm is one of the most popular supervised learning method basing on the concept of maximal margin hyperplane [32]. The hyperplane separates training samples with 2 different labels, from which both positive and negative categories have the largest distances. Multi-class problem will be transformed into binary class problem such as one-against-one or one-against-all. Kernels approach will be used to construct nonlinear decision boundary if the data is not linearly separable. We used Radial Basis Function kernel as follows: $K\left(x_{i},x_{j}\right)=e^{-||x_{i}-x_{j}|{|}^{2}/c}$, where c > 0, c is a scalar.

### k-nearest neighbor algorithm

k-nearest neighbor algorithm (kNN) is a nonparametric method of supervised classification, basing on distance function *d*(*x_q_*, *x_i_*), such as Euclidean distance. The original data was preprocessed so that the values of each feature in the data have zero mean and unit variance [33]. The distances of k nearest neighbors were weighted and labeled to refine the model, the improved kNN algorithm is depicted as: $F\left(x_{q}\right)=\underset{v\in V}{\text{arg}\text{max}}\overset{k}{\underset{i=1}{\sum }}w_{i}\delta \left(v,f\left(x_{i}\right)\right)$, where $w_{i}=\frac{1}{d{\left(x_{q},x_{i}\right)}^{2}}$; *f*(*x_i_*) is the label of the *i*th sample; and *δ*(a,b) = 1 when a = b, otherwise *δ*(a,b) = 0. *F*(*x_q_*) was assigned to *f*(*x_i_*) when the distance between *x_q _*and *x_i _*become zero [34]. Cross validation method were used to determine the k values.

### k means clustering method

k means clustering is an unsupervised classification method for finding clusters and cluster centers. The method works in three steps: (1) Select the first kth samples as the seed mean; (2) Classify samples according to the nearest mean value; (3) End the loop when there is no change in the mean values. We used Euclidean distance as distance function. The program can be downloaded from http://people.revoledu.com/kardi/tutorial/kMean/matlab_kMeans.htm. Each feature was standardized to mean 0 and variance 1 in the training before the performance of k means clustering [33].

### Mutual information

Mutual information measures the mutual dependence between two variables based on information theory. The mutual information of two continuous variables × and Y is defined as: $\text{I(x,y)}=\int \int \text{p(x,y)}\text{log}\frac{\text{p(x,y)}}{\text{p(x)p(y)}}dxdy$, Where p(x) and p(y) are the frequencies of appearances, and p(x, y) is the joint probabilistic density.

In case of discrete variables, mutual information is defined as: $I\left(X;Y\right)=\underset{y\in Y}{\sum }\underset{x\in X}{\sum }p\left(x,y\right)\text{log}\left(\frac{p\left(x,y\right)}{p\left(x\right)p\left(y\right)}\right)$.

We sorted the mean values of each feature class, computed average values of each adjacent values, and discretized each features according to the average values, then calculated the mutual information. Datasets with mutual information below 0.03 threshold were considered as noisy data, thus mutual information method was used instead of Fisher statistic method at feature deletion step.

To measure classification ability on noisy data, we discretized features according to median value of classes for each feature, then compute mutual information.

### Minimum Redundancy Maximum Relevance

Minimum Redundancy Maximum Relevance (mRMR) method is widely used for feature selection such as gene selection [35]. The Maximum Relevance is defined as: $\underset{S}{\text{max}}\frac{1}{\left|S\right|}\overset{}{\underset{g_{i}\in S}{\sum }}I\left(g_{i};c\right)$, Where I(x, y) is mutual information of two variables × and y, S is the selected vector set, g is a feature of S, and c is the class label.

The Minimum Redundancy is defined as: $\underset{S}{\text{min}}\frac{1}{|S{|}^{2}}\overset{}{\underset{g_{i},g_{j}\in S}{\sum }}I\left(g_{i};g_{j}\right)$.

The mRMR feature set is obtained by optimizing the Maximum Relevance and Minimum Redundancy simultaneously. Optimization of both conditions requires combining them into a single criterion function. In this paper, the m-th feature was selected according to the value of Maximum Relevance divided by Minimum Redundancy [20]: $\underset{g_{i}\in G-S_{m-1}}{\text{max}}\frac{I\left(g_{i};c\right)}{\frac{1}{m-1}\overset{}{\underset{g_{i}\in S_{m-1}}{\sum }}I\left(g_{i};g_{j}\right)}$.

mRMR method need to discrete training data before running, so considering sparse discrete of the data, we assign 1 for features with expression information and 0 for features without expression. The mRMR program can be downloaded from web site: http://penglab.janelia.org/proj/mRMR/

### Kruskal-Wallis test

Kruskal-Wallis test is a non-parametric method for testing whether samples originate from the same distribution [23]. The test assumes that all samples from the same group have the same continuous distribution, and they are mutually independent. In this study, Kruskal-Wallist test was used to rank features. The program can be downloaded from http://featureselection.asu.edu/algorithms/fs_sup_kruskalwallis.zip.

### Information Gain

Information Gain measures the classification ability of each feature with respect to the relevance with the output class, which is defined as Information Gain = H(S)-H(S|x) [27], $\text{H}\left(\text{S}\right)=-\underset{s\in S}{\sum }p\left(s\right){\text{log}}_{2}\left(p\left(s\right)\right)$, $H\left(S|\text{x}\right)=-\underset{x\in X}{\sum }p\left(x\right)\underset{s\in S}{\sum }p\left(s|x\right){\text{log}}_{2}\left(p\left(s|x\right)\right)$, where S and × are features. When measuring the mutual relation between the extracted features and the class, Information Gain is also known as mutual information [21]. We assigned 1 to features with expression information and 0 to features without expression, and ranked the Information Gain values; the larger the value, the more important is the feature.

### χ^2 ^statistic

The Chi-squared (χ^2^) statistic uses theχ^2 ^statistic to discretize numeric attributes and achieves feature selection via discretization [22]. Theχ^2 ^value is defined as $\chi^{2}\text{ = }\overset{c}{\underset{i=1}{\sum }}\overset{k}{\underset{j=1}{\sum }}\frac{{\left(A_{ij}-E_{ij}\right)}^{2}}{E_{ij}}$, where c is the number of intervals, k is the number of classes, *A_ij _*is the number of samples in the *i*th interval and the *j*th class, *M_i _*is the number of samples in the *i*th interval, *B_j _*is the number of samples in the *j*th class, N is the total number of samples, and $E_{ij}=\frac{M_{i}B_{j}}{N}$. We assigned 1 for features with expression information and 0 for features without expression, and sorted theχ^2 ^statistic values, the lager the value, the more important is the feature.

## Abbreviations

FMS: Feature Merging and Selection algorithm; PCA: Principal Component Analysis; LDA: Linear Discriminant Analysis; HAP: hospital-acquired pneumonia; CAP: community-acquired pneumonia; kNN: K-nearest neighbor algorithm; SVM: Support vector machine; mRMR: Minimum Redundancy Maximum Relevance.

## Competing interests

The authors declared that they have no competing interests.

## Authors' contributions

YW performed algorithm design and wrote the manuscript. YZ, ZL and YZ collected the data. YL and XG designed and sponsored the study. HS contributed and edited the manuscript. All authors read and approved the manuscript.

## Supplementary Material

Additional file 1 **Supplementary materials, pdf format**. Click here for file
