# Supplementary material for: An improved dimensionality reduction method for meta-transcriptome indexing based diseases classification
Source: BMC Syst Biol. 2012 Dec 17;6(Suppl 3):S12. doi: 10.1186/1752-0509-6-S3-S12 (PMC3524076; doi:10.1186/1752-0509-6-S3-S12)
Supplement: Additional file 1 — Supplementary materials, pdf format. [file 1752-0509-6-S3-S12-S1.pdf]

Supplementary materials

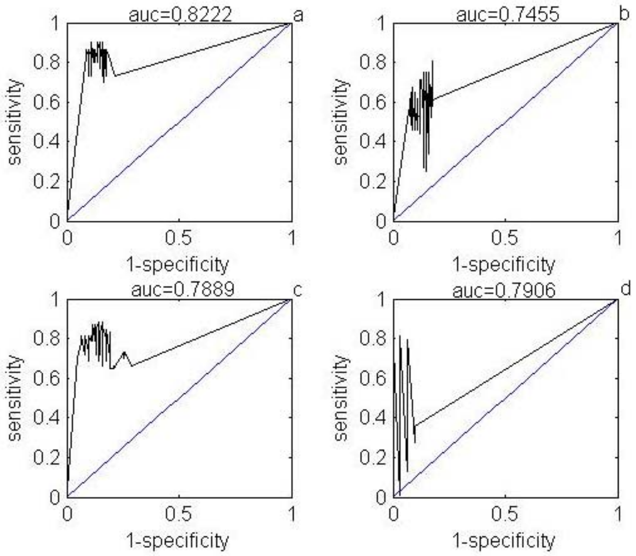

Figure S1: ROC curves of feature merging step of FMS algorithm for normal samples in 3-class problem (a), CAP samples in 3-class problem (b), HAP samples in 3-class problem (c) and pneumonia samples (CAP and HAP) in 2-class problem (d).

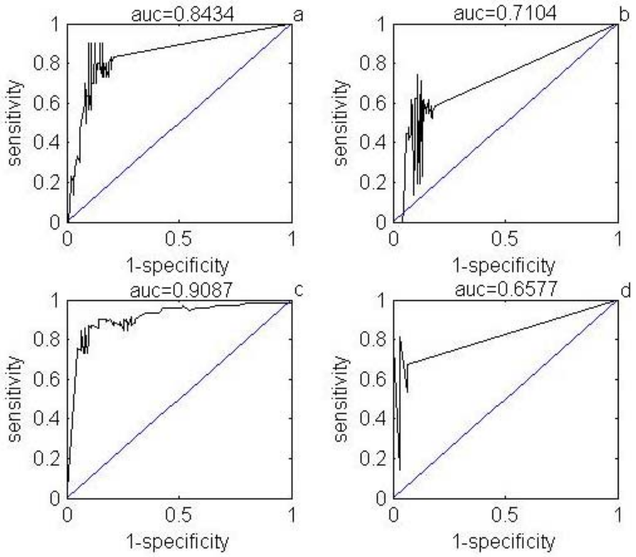

Figure S2: ROC curves of feature deletion step of FMS algorithm for normal samples in 3-class problem (a), CAP samples in 3-class problem (b), HAP samples in 3-class problem (c) and pneumonia samples (CAP and HAP) in 2-class problem (d).

Table S1: Microbiota signatures in 3-class and 2-class problem. Absolute weights indicate contribution levels of the microbiota for the classification, large weight means high level of importance on the classification. Features of same weight signs are positive correlated in the sense of their rRNA's expression levels and vice versa.

3-class problem		2-class problem	
Microbiota	Weight	Microbiota	Weight
Shuttleworthia	0.409654483	Acidaminococcus	0.9375742
Mobiluncus	-0.338191098	Eubacterium	0.315648406
Peptococcus	0.335372548	Enhydrobacter	0.104247946
Deinococcus	-0.320586995	Peredibacter	-0.099991176
Centipeda	-0.270139573	Aquabacterium	0.013513469
Finegoldia	-0.264143868	Fusobacterium	-0.009490509
Anaeroglobus	-0.192251314	Cardiobacterium	0.008837628
Enhydrobacter	0.190325525	Selenomonas	-0.007631117
Gemmatimonas	-0.188063703	Mycoplasma	0.005059734
Mogibacterium	0.184186615	Leptotrichia	-0.004495709
Filifactor	-0.170199257		
Slackia	0.170085945		
Methylibium	-0.160199342		
Methylobacterium	0.13737352		
Salinivibrio	0.118884871		
Jeotgalicoccus	-0.110401787		
Kingella	-0.102107897		
Atopobium	-0.101942619		
Acidaminococcus	0.095202428		
Alcaligenes	0.092685765		
Bradyrhizobium	-0.091781792		
Xanthobacter	0.088064469		
Hydrocarboniphaga	0.087916384		
Comamonas	-0.082752919		
Bdellovibrio	-0.066375238		
Kluyvera	-0.057522578		
Anaerosporobacter	-0.053649555		
Leptotrichia	-0.04333114		
Stenotrophomonas	-0.036306241		
Nitrospira	0.032863576		
Peptostreptococcus	-0.029646383		
Megasphaera	-0.029175943		
Parvimonas	-0.023960797		
Fusobacterium	-0.022136506		
Hydrogenophaga	-0.019570124		
Cardiobacterium	0.018299338		

Gemella	-0.01268344		
Streptococcus	-0.011958471		
Lactobacillus	-0.01155858		
Pseudomonas	-0.010848654		
Staphylococcus	-0.010048541		
Enterococcus	-0.009560343		
Acinetobacter	-0.009285258		
Prevotella	-0.009036361		
Peredibacter	-0.007426782		

Table S2: Classification ability on dental decay data from saliva samples.

Method	Error rate		Dimension	Feature number	Note
	On training data	On test data			
svm/FMS	0.2583	0.4118	46	65	
svm/mRMR	0.2806	0.5294	55	55	
svm/Kruskal Wallis	0.2861	0.5882	8	8	
Svm/InformationGain	0.3	0.5294	58	58	
svm/ χ2 statistic	0.3028	0.5882	38	38	
svm	0.3917	0.4118	70	70	
kNN/FMS	0.3278	0.4118	6	6	k=1
kNN/mRMR	0.3056	0.4808	58	58	k=1
kNN/Kruskal Wallis	0.2361	0.4118	1	1	k=7
kNN/InformationGain	0.3056	0.4118	60	60	k=6
kNN/ χ2 statistic	0.3056	0.4706	59	59	k=3
kNN	0.375	0.5162	70	70	k=3

Table S3: Classification ability on dental decay data in from plaques samples.

Method	Error rate		Dimension	Feature number	Note
	On training data	On test data			
svm/FMS	0.3385	0.4444	13	65	
svm/mRMR	0.3925	0.6667	17	17	
svm/KruskalWallis	0.4782	0.4444	72	72	
svm/InformationGain	0.421	0.5	22	22	
Svm/ χ2 statistic	0.4183	0.6111	36	36	
svm	0.4782	0.4444	72	72	
kNN/FMS	0.3452	0.4444	21	23	k=1
kNN/mRMR	0.3544	0.5	71	71	k=1
kNN/KruskalWallis	0.4016	0.5	72	72	k=1
kNN/InformationGain	0.3321	0.5	71	71	k=1
kNN/ χ2 statistic	0.3321	0.5	71	71	k=1
kNN	0.4016	0.5	72	72	k=1
